# Does acute isolated sphenoidal sinusitis meet the criteria of the recent acute sinusitis guidelines, EPOS2020?

**DOI:** 10.1007/s00405-023-08405-y

**Published:** 2024-01-16

**Authors:** Raed Farhat, Ashraf Khater, Nidal El Khatib, Majd Asakly, Aviva Ron, Alaa Safia, Marwan Karam, Saqr Massoud, Yaniv Avraham, Shlomo Merchavy

**Affiliations:** 1 Department of Otolaryngology, Head and Neck Surgery Unit, Rebecca Ziv Medical Center, Postal code – 1028- Golan Heights, Buqata, Safed, Israel; 2 Bar-Ilan University’s Azrieli Faculty of Medicine, Safed, Israel; 3 Research Wing, Rebecca Ziv Medical Center, Safed, Israel; 4 Independent Consultant, Safed, Israel

**Keywords:** ISS: Isolated sphenoidal sinusitis, ARS: Acute rhinosinusitis, ABRS: Acute bacterial rhinosinusitis, CRS: Chronic rhinosinusitis, ED: Emergency department, EPOS: European Position Paper on Rhinosinusitis

## Abstract

**Introduction:**

Isolated sphenoidal sinusitis (ISS) is a rare disease with non-specific symptoms and a potential for complications. Diagnosis is made clinically, endoscopically, and with imaging like CT scans or MRIs. This study aimed to evaluate if ISS meets the EPOS 2020 criteria for diagnosing acute rhinosinusitis and if new diagnostic criteria are needed.

**Materials and methods:**

The study analyzed 193 charts and examination records from 2000 to 2022 in patients diagnosed with isolated sphenoidal sinusitis at the Ziv Medical Center in Safed, Israel. Of the 193, 57 patients were excluded, and the remaining 136 patients were included in the final analysis. Patients were evaluated using Ear, Nose and Throat (ENT), neurological and sinonasal video endoscopy, radiological findings, demographic data, symptoms and signs, and laboratory results. All these findings were reviewed according to the EPOS 2020 acute sinusitis diagnosis criteria and were analyzed to determine if ISS symptoms and signs fulfilled them.

**Results:**

The patients included 40 men and 96 women, ranging in age from 17 to 86 years (mean ± SD, 37 ± 15.2 years). A positive endoscopy and radiography were encountered in 29.4%, and headache was present in 98%; the most common type was retro-orbital headache (31%). The results showed that there is no relationship between the symptoms of isolated sphenoidal sinusitis and the criteria for diagnosing acute sinusitis according to EPOS 2020.

**Conclusion:**

ISS is an uncommon entity encountered in clinical practice with non-specific symptoms and a potential for complications. Therefore, the condition must be kept in mind by clinicians, and prompt diagnosis and treatment must be initiated. This kind of sinusitis does not fulfill the standard guidelines for acute sinusitis diagnosis criteria.

## Introduction

Isolated sphenoidal sinusitis (ISS) is a relatively uncommon disease. Among patients with rhinosinusitis, isolated sphenoiditis is diagnosed in 1–3% of cases [1, 2].

According to the literature, the most common cause of sphenoid sinus lesions is inflammation, as found in up to 65%–72% of cases, followed secondly by neoplasms, which account for 18% (in benign) and 10.9% (in malignant) of the cases, respectively [3].

Because of its deep-seated anatomy, this sinus does not usually present with nasal symptoms such as nasal obstruction or rhinorrhea. The most common symptom is headache, which is prevalent in about 98% of inflammatory lesions [4].

In the majority of cases, symptoms do not arise in the early stages of the disease or are non-specific, and some cases may subsequently be referred to the otolaryngology department after significant progression of the disease [5]. Diagnosis is made clinically, endoscopically, and with the help of current imaging techniques such as CT scans or MRIs. All three previously mentioned modalities, especially endoscopic examination and imaging techniques, increased the likelihood of diagnosing such a disease. A computed tomographic scan (CT) is used as the first choice of investigation [6].

In the study by Berg and Carenfelt [7], the presence of two or more findings (purulent rhinorrhea and local pain with unilateral predominance, pus in the nasal cavity, and bilateral purulent rhinorrhea) showed 95% sensitivity and 77% specificity for the diagnosis of acute bacterial rhinosinusitis (ABRS). The estimated parameters of endoscopy are superior to those of radiography, with a sensitivity of 80% and a specificity of 94% [8].

Acute bacterial sinusitis is suspected in patients whose upper respiratory tract infection has persisted beyond 10–14 days. [9, 10]. Prominent symptoms in adults include nasal congestion, purulent rhinorrhea, facial-dental pain, postnasal drainage, headaches, and coughs [11]. Although all of these symptoms are non-specific [12, 13], a history of persistent purulent rhinorrhea and facial pain appears to have some correlation with an increased likelihood of bacterial disease. [14, 15]

The European Position Paper on Rhinosinusitis and Nasal Polyps 2020 (EPOS2020) is the latest in a series of evidence-based position papers that aim to provide clear evidence-based recommendations and integrated care pathways in ARS and CRS [16].

Acute rhinosinusitis (ARS): management as per EPOS 2020: Acute rhinosinusitis in adults is characterized by two or more symptoms, one of which should be either: nasal blockage, swelling, or congestion; nasal discharge (anterior or posterior nasal drip); ± facial pain or pressure; ± reduction or loss of smell (cough in children); and a duration of < 12 weeks [17] (Table 3).

Our study attempted to evaluate cases of isolated acute sphenoidal sinusitis over a 22-year period who were admitted to the Ziv Medical Center emergency department (ED).

The diagnosis was based on clinical features and CT findings with medical consensus.

The goal of this study is to describe the different features, the initial symptoms, and the common practice findings between both neurologists and otolaryngologists.

Our main questions were:

- What are the clinical presentations and symptoms of acute isolated sphenoidal sinusitis?
- Who was the first physician to examine the patient in the ED?
- What are the common practice findings between both neurologists and otolaryngologists?
- Does ISS meet the criteria of EPOS 2020 [17] for diagnosing acute rhinosinusitis? Analyzing retrospectively patients' symptoms and endoscopic findings with statistical analysis
- Do we need new diagnostic criteria for this rare entity apart from the EPOS 2020 criteria?

This study is the first one in the literature to analyze such a diagnostic dilemma compared to the most updated evidence-based acute sinusitis diagnosis guidelines.

## Materials and methods

We analyzed 193 charts and examination records from 2000 to June 2022 in patients who were admitted to the ED and diagnosed with isolated sphenoidal sinusitis in Ziv Medical Center, Israel. Of the 193, 57 patients were excluded, and the remaining 136 patients were included in the study.

Our primary study inclusion criteria were collected according to: 1. Clinical presentation: For patients who presented to the ED with an acute ISS diagnosis involving only the unilateral sphenoid sinus, we reviewed patients’ complaints with detailed descriptions of headaches (type, severity, frequency, duration) and other presenting symptoms (nasal congestion, rhinorrhea, facial pain, loss of smell, cough, visual disturbances, etc.). 2. Radiologic findings (CT imaging): detailed assessment of sphenoid sinus opacity or abnormalities; documentation of any abnormality such as mucosal thickening, fluid accumulation, or bony erosion. 3. Endoscopic examination findings (such as the presence of pus, polyps, or mucosal abnormalities).

In each case, we evaluated the following:

1) Demographic data (age, gender, major diagnosis, medical history).

2) Patient symptoms and signs in the medical record: nasal blockage, obstruction, congestion or nasal discharge; anterior or posterior nasal drip; reduction or loss of sense of smell; fever > 38 °C; facial pain or pressure; headache type (frontal, retro-orbital, trigeminal, vertex, temporal, occipital); dizziness; blurred vision; syncope; etc. The data were collected by both the otolaryngologist and neurological documentation.

All these findings were reviewed according to EPOS 2020 diagnosis criteria and analyzed to see if the criteria were fulfilled.

3) Endoscopic signs of nasal polyps and/or mucopurulent discharge primarily from the superior meatus.

4) CT changes: mucosal thickness; the presence or absence of an air–fluid level and air bubbles in the isolated sphenoidal sinus; and whether the lesion was round or expansive, completely opaque, irregularly shaped, and showed calcification on CT and/or the flow void sign on MRI—if the patients completed an MRI scan. Based on imaging, we categorized the cases as follows: inflammation, fungal disease, mucocele, cysts, and unclassifiable.

5) Who was the first physician who examined the patient in the ED?

6) Neurological findings: physical examination, comparing the patient’s history with ENT history and combining these, pathological findings, and the patient's results if they underwent a lumbar puncture.

7) Laboratory results: leukocytes, CRP.

Patients were excluded from the study if: 1) they had ISS involving the bilateral sphenoid sinuses or other sinuses; 2) prior skull base surgery or transnasal pituitary surgery; 3) brain tumor (including suspected cases); 4) disease of the sphenoid bone (including suspected cases); 5) acute head trauma; 7) suspected chronic lesion; 8) patients who were treated in the first 10 days after symptom onset; or 9) if there was a lack of data or follow-up.

The patients who were excluded were: 8 patients had bilateral sphenoid sinuses; 2 had prior skull base surgery; 1 had a brain tumor; 4 had a disease of the sphenoid bone; 5 came with acute head trauma; 15 patients were treated in the first 10 days after symptom onset; and 11 had a lack of data or follow-up. Five patients were diagnosed with benign or malignant pathologies: four with fungal sinusitis and two with mucocele. We started the analysis with the ED's first visit.

Patients were evaluated in most cases first by objective neurological examination (headache as a major complaint), objective ear, nose, and throat examination, and by nasal-sinus video endoscopy, with or without radiological diagnosis in the ED. If the patient did not undergo CT, this was done after admission to the hospital department. At the time of the diagnosis, there was no involvement of other sinuses. The diagnosis was confirmed by CT of the paranasal sinuses and nasal cavities in axial and coronal sections, without contrast fluid.

In some cases, MRI was used when there was a neurological pathology, possible bone erosion of the sinus wall, the presence of a cerebrospinal fluid leak, and visual changes.

Patients were admitted mostly in the ENT department and sometimes in the neurology department because severe headaches were the major complaint and suspected meningitis had to be ruled out.

Admitted patients who were diagnosed with isolated sphenoiditis without complications were treated with intravenous antibiotics ceftriaxone and clindamycine for 3–6 days associated with corticosteroids. As mentioned above, admitted patients who were diagnosed with isolated sphenoiditis without complications were treated with intravenous antibiotics.

Only 3 patients were treated with endoscopic surgical procedures because of the worsening or maintenance of the severity of symptoms during the treatment or within 6 weeks after it.

Benign or malignant pathologies were excluded.

### Statistical analysis

Data were analyzed using the statistical package for social sciences (SPSS) version 21 (SPSS Inc.; Chicago, IL, USA). The data were described by the mean standard deviation (SD) and frequency. Normal and non-normal distribution variables were compared using a one-sample *t-*test. The normality of the variables was checked by the one-sample Kolmogorov–Smirnov test.

A one-sample t-test compares the mean of a sample to a population mean. When only a sample standard deviation is known, a t-test should be used. If the calculated t-value is less than the critical t-value, the null hypothesis (hypothesis of no difference) should not be rejected, with no significant difference between the sample mean and population mean declared. However, if the calculated t-value is larger than the critical t-value, the null hypothesis should be rejected, with a significant difference in means declared.

The null hypothesis (*H*0) and two-tailed alternative hypothesis (*H*1) of the one-sample *T* test can be expressed as:

*H*0: µ = µ0 ("the population mean is equal to the [proposed] population mean").

*H*1: µ ≠ µ0 ("the population mean is not equal to the [proposed] population mean").

where µ is the "true" population mean and µ0 is the proposed value of the population mean.

## Results

There were 40 men and 96 women, ranging in age from 18 to 86 years (mean ± SD, 37 ± 15.2 years) (Table 1).

**Table 1:** Demographics

Total Patients	136		
Gender	Number of patients	Age Range (years)	Mean Age (± SD) (years)
Men	40	18–80	40 +—15.07
Women	96	18–77	36 +—15.11

Symptoms lasted for a mean ± SD of 15.6 ± 9.3 days. Of the 136 patients, 34 were given antibiotics by the community physician before referral to the hospital.

Headache was the major complaint that presented in 98% of our series. Patients reported a major complaint of several types of headaches; the most common type was retro-orbital headache (31%), vertex headache (28%), and frontal headache (20%). 14 patients had postnasal drip (10%), 32 patients had nasal obstruction (23%), and 12 had fever (8%). There were 2 cases of a decrease in consciousness level (Table 2).

**Table 2:** Symptoms and complaints

Symptoms	Percentage of Patients
Headache	98%
Retro-orbital headache	31%
Vertex headache	28%
Frontal headache	20%
Postnasal drip	10%
Nasal obstruction	23%
Fever	8%
Decrease in consciousness level	2 cases

Positive endoscopy and radiography findings were found in 39 patients (29.4%). Video endoscopy of these patients revealed signs of inflammation, characterized by mucosal edema and mucopurulent discharge arising from the sphenoethmoidal recess.

Patients who presented with acute isolated sphenoidal sinusitis were classified into subgroups according to the diagnostic criteria of acute sinusitis based on EPOS 2020, which is known as the latest series of guidelines on rhinosinusitis from an international cohort of experts in the field (Table 3). If the p value is less than 0.05 and the difference is statistically significant, the null hypothesis is therefore rejected. This illustrates that there is no relationship between two variables: the symptoms of isolated sphenoidal sinusitis and the criteria for diagnosing acute sinusitis according to EPOS 2020 (Table 4). We also used Pearson’s r to measure the strength of the relationship between isolated sphenoidal sinusitis symptoms and the EPOS 2020 criteria, as shown in Table 5, which revealed that there is no correlation.

**Table 3:** Acute rhinosinusitis (ARS) diagnosis as per EPOS 2020 Acute rhinosinusitis in adults [17]

2.1.2. Clinical definition	
2.1.3.1. Acute rhinosinusitis (ARS) in adults	Acute rhinosinusitis in adults is defined as: sudden onset of two or more symptoms, one of which should be either nasal blockage/obstruction/congestion or nasal discharge (anterior/posterior nasal drip): • ± facial pain/pressure, • ± reduction or loss of smell for < 12 weeks see reference 17

**Table 4:** We tested the significance by evaluating t-statistics and comparing it with critical value read from t-distribution table at 0.05 significance level [18–20]

	N	Mean	Std. Deviation	Std. Error Mean	t	df	Sig. (2-tailed)	Mean Difference	95% Confidence Interval of the Difference	
									Lower	Upper
Facial Pressure	136	.12	.323	.028	−22.804	135	.000	−.632	−.69	−.58
Purulent Discharge in nasal endoscopy	136	.29	.454	.039	−5.478	135	.000	−.213	−.29	−.14
Posterior Nasal Drip PND	136	.08	.274	.023	−26.383	135	.000	−.619	−.67	−.57
Nasal Obstruction with anterior purulent nasal discharge	136	.22	.416	.036	−17.637	135	.000	−.629	−.70	−.56
reduction or loss of smell	136	.07	.250	.021	−24.951	135	.000	−.534	−.58	−.49
CRP	136	12.56	20.872	1.790	3.665	135	.000	6.559	3.02	10.10
Criteria EPOS 2020	136	.24	.430	.037	−13.751	135	.000	−.507	−.58	−.43

If the t-statistics value is greater than the critical value at 0.05, the null hypothesis can be rejected. This would mean that there is enough evidence to support the alternate hypothesis that there is no relationship between two variables—the symptoms of isolated sphenoidal sinusitis and criteria of diagnosing acute sinusitis according to EPOS 2020

**Table 5:** We used Pearson’s r to measure the strength of the relationship between isolated sphenoidal sinusitis symptoms and EPOS 2020 Criteria as shown in which reveals that there is no correlation except "headache" which is not a criterion in EPOS 2020 Guidelines

	Distribution Percent (%) among patients with ISS		CRITERIA EPOS 2020
Facial pressure	**11.8%**	Pearson Correlation	.539^**^
		Sig. (2-tailed)	0.000
		N	136
Nasal obstruction with anterior purulent nasal discharge	**22.1%**	Pearson Correlation	.940^**^
		Sig. (2-tailed)	0.000
		N	136
Posterior Nasal Drip (PND)	**8.1%**	Pearson Correlation	.524^**^
Headache			
		Sig. (2-tailed)	0.000
		N	136
Headache Retro-orbital	**64.7%**	Pearson Correlation	0.156
		Sig. (2-tailed)	0.069
		N	136
Headache vertex	**69.1%**	Pearson Correlation	-0.118
		Sig. (2-tailed)	0.170
		N	136
Headache occipital	**87.5%**	Pearson Correlation	0.045
		Sig. (2-tailed)	0.600
		N	136
Headache trigeminal distribution	**10.3%**	Pearson Correlation	-0.135
		Sig. (2-tailed)	0.116
		N	136
Headache frontal	**77.9%**	Pearson Correlation	-0.012
		Sig. (2-tailed)	0.894
		N	136
Headache temporal region	**7%**	Pearson Correlation	-0.049
		Sig. (2-tailed)	0.573
		N	136
Reduction or loss of smell	**6.6%**	Pearson Correlation	.401^**^
		Sig. (2-tailed)	0.000
		N	136
Purulent Discharge in nasal endoscopy	**28.7%**	Pearson Correlation	.703^**^
		Sig. (2-tailed)	0.000
		N	136
CRITERIA EPOS 2020		Pearson Correlation	1
		Sig. (2-tailed)	
		N	136
High CRP	**36.3%**	Pearson Correlation	0.128
		Sig. (2-tailed)	0.140
		N	135

** Correlation is significant at the 0.01 level (2-tailed). * Correlation is significant at the 0.05 level (2-tailed)

c. Cannot be computed because at least one of the variables is constant

A lumbar puncture was performed in 23 patients to rule out meningitis; no one had meningitis in our series, and the results are shown in Table 6**.**

**Table 6:** Cerebrospinal fluid findings from the lumbar puncture among 23 patients with isolated sphenoidal sinusitis

Patients who undergone LP	WBC—White Blood Cells (/mm^3^)	CSF Glucose (mg/dl)	Protein (mg/dl)	Gram Stain	CSF Culture
P1	0	59	15–45	Negative	Negative
P2	0	43	15–45	Negative	Negative
P3	0	55	15–45	Negative	Negative
P4	2	63	15–45	Negative	Negative
P5	0	54	15–45	Negative	Negative
P6	4	44	15–45	Negative	Negative
P6	0	70	15–45	Negative	Negative
P7	0	62	15–45	Negative	Negative
P8	0	57	15–45	Negative	Negative
P9	0	49	15–45	Negative	Negative
P10	0	78	15–45	Negative	Negative
P11	0	65	15–45	Negative	Negative
P12	0	45	15–45	Negative	Negative
P13	0	56	15–45	Negative	Negative
P14	0	58	15–45	Negative	Negative
P15	2	73	15–45	Negative	Negative
P16	0	70	15–45	Negative	Negative
P17	0	71	15–45	Negative	Negative
P18	0	49	15–45	Negative	Negative
P19	4	62	15–45	Negative	Negative
P20	0	74	15–45	Negative	Negative
P21	3	57	15–45	Negative	Negative
P22	0	43	15–45	Negative	Negative
P23	8	6	15–45	Negative	Negative
P = Patient Normal Values: WBC < 5/mm^3^; Protein,15–45 mg/dl; Glucose > 2/3 of serum, 40–80 mg/dl; CSF: Cerebrospinal Fluid					

## Discussion

Based on the results of this study and the literature, we recommend a new diagnostic strategy for patients with isolated unilateral acute sphenoidal sinusitis.

There is a lack of consensus on how we diagnose acute isolated sphenoidal sinusitis (AISS), with wide variation in diagnosis across guidelines. This lack of uniformity in disease definition stems in part from an incomplete understanding of disease pathobiology, phenotypic heterogeneity, and natural history.

Unlike other diseases, where definitions are centered around a specific diagnostic test, AISS is not only a clinical syndrome for which we rely on a constellation of symptoms, signs, and other manifestations. We also often need a radiological diagnosis combined with clinical features.

In this study, we reviewed gaps between guidelines and existing diagnostic tools. Ebell et al. concluded in a meta-analysis [20] that acute rhinosinusitis as diagnosed by any reference standard is significantly less likely in patients without any nasal discharge, without a complaint of purulent nasal discharge, and with normal transillumination. According to our results, this conclusion does not include isolated sphenoidal sinusitis.

In another study by Berg and Carenfelt [21], the presence of two or more findings (purulent rhinorrhea and local pain with unilateral predominance, pus in the nasal cavity, and bilateral purulent rhinorrhea) showed 95% sensitivity and 77% specificity for the diagnosis of ABRS.

Inflammatory sinusopathy does not mean an infectious presentation; that is, we should also consider the viral infection in the first days of the disease. To classify acute rhinosinusitis, we should take into consideration the duration of the symptoms, so in this study, all the patients who had symptoms before 10 days of treatment were excluded. According to EPOS 2012, 2020, [17 and 22], acute bacterial rhinosinusitis is classified as having symptoms lasting longer than 10 days.

The diagnosis of acute rhinosinusitis is mainly clinical. In the practice of medicine, it refers to the clinical presentation of a particular patient at a time when an accurate and correct diagnosis may be difficult to make.

In the clinical evaluation of a patient with a diagnostic dilemma, a physician has at his or her disposal numerous diagnostic technologies, including laboratory tests, imaging, and even interventional procedures.

Lew et al. [23]reported 30 patients with infectious sphenoid sinusitis (15 acute cases, 15 chronic cases). The main symptom of their patients was a headache that radiated to the occipital region or pain in the trigeminal nerve distribution. Similar to our results, the authors agreed that CT was the most useful method for the diagnosis of sphenoid sinus disease.

After reviewing 122 individuals, Fooanant et al. [24]came to the conclusion that sphenoid sinus illness could not be completely ruled out by a routine endoscopic examination. The most prevalent pathology across all investigations was infection or inflammation. The majority of investigations found that bacterial sphenoiditis was the most prevalent lesion in this group. Because of its high intensity and intractability, the attending physicians asked for imaging tests such as a brain CT scan. Headaches were the most prevalent symptom in their study, appearing in 63% of participants.

The sphenoid sinus is uncommonly involved solely by inflammatory disease and tumors [25]. Lew et al. [23] suggested an incidence of 2.7% for isolated sphenoid disease; however, Hnatuk et al. [26] estimated it to be less than 1% of all sinus disease.

Epos 2020 defined the alarm symptoms as follows: periorbital edema or erythema; displaced globe; double vision; ophthalmoplegia; reduced visual acuity; severe headache; frontal swelling; signs of sepsis; signs of meningitis; and neurological signs requiring an immediate referral.

The main limitation of the study is to consider only isolated bacterial sinusitis. This model is not applicable to other types of sphenoiditis.

The study’s strengths are that it describes a rare cause of sinusitis that was identified by a multidisciplinary team, including neurologists with lumbar puncture results for the first time in the literature, in addition to otolaryngologists findings, and that there was a lengthy follow-up of those patients following treatment. Finally, it can help to develop a new method for identifying this uncommon type of sinusitis, particularly since it does not fit the guidelines for acute sinusitis diagnosis.

## Conclusion

ISSD is an uncommon entity in clinical practice with non-specific symptoms and a potential for complications. The most common symptom is headache. The neurologist may be the first doctor to check patients in the emergency department. Therefore, the condition must be kept in mind by clinicians, prompt diagnosis with an otolaryngologist must be made, and treatment must be initiated. This kind of sinusitis does not fulfill the standard guidelines for acute sinusitis diagnosis criteria. For the diagnosis of ISS, we advise creating a chain of diagnostic algorithms. Adhering to such a protocol can help patients and doctors feel more secure about the diagnosis and treatment strategy while also being cost-effective and requiring fewer invasive procedures.

## Data Availability

The data that support the findings of this study are available from the corresponding author, [RF], upon reasonable request.
